# Supplementary material for: Low Serum Branched-chain Amino Acid and Insulin-Like Growth Factor-1 Levels Are Associated with Sarcopenia and Slow Gait Speed in Patients with Liver Cirrhosis
Source: J Clin Med. 2020 Oct 10;9(10):3239. doi: 10.3390/jcm9103239 (PMC7600046; doi:10.3390/jcm9103239)
Supplement: Supplementary file 1 [file jcm-09-03239-s001.pdf]

Low Serum Branched-chain Amino Acid and Insulin-Like Growth Factor-1 Levels Are Associated with Sarcopenia and Slow Gait Speed in Patients with Liver Cirrhosis

Chisato Saeki [1,2,*], Tomoya Kanai, MD [1,2], Masanori Nakano [1,2], Tsunekazu Oikawa [1], Yuichi Torisu [1,2], Masayuki Saruta [1], and Akihito Tsubota [3,*]

Supplementary Materials:

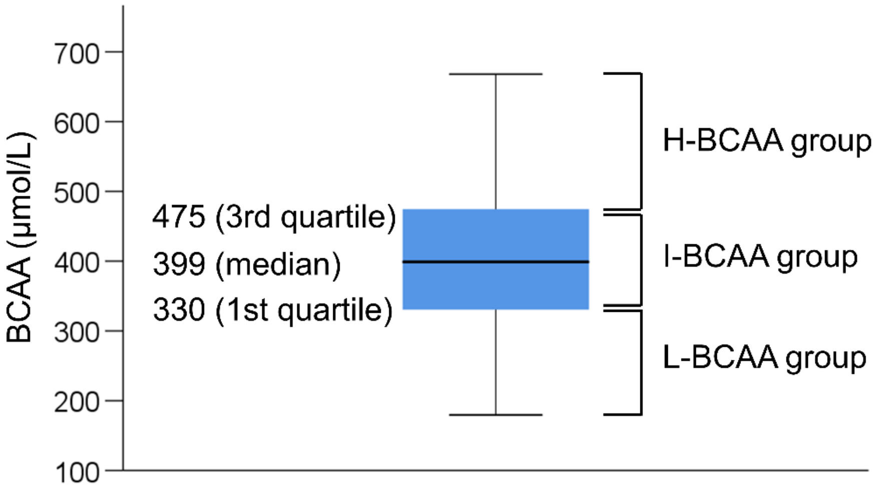

Figure S1. Classification based on baseline serum branched-chain amino acid (BCAA) levels. The median (interquartile range) of the BCAA levels was 399 (330–475) µmol/L. The 192 patients were classified into three groups: (1) low (L)-BCAA group with BCAA levels ≤330 µmol/L (first quartile); (2) intermediate (I)-BCAA group with BCAA levels between 330 and 475 µmol/L (third quartile); and (3) high (H)-BCAA group with BCAA levels ≥475 µmol/L.

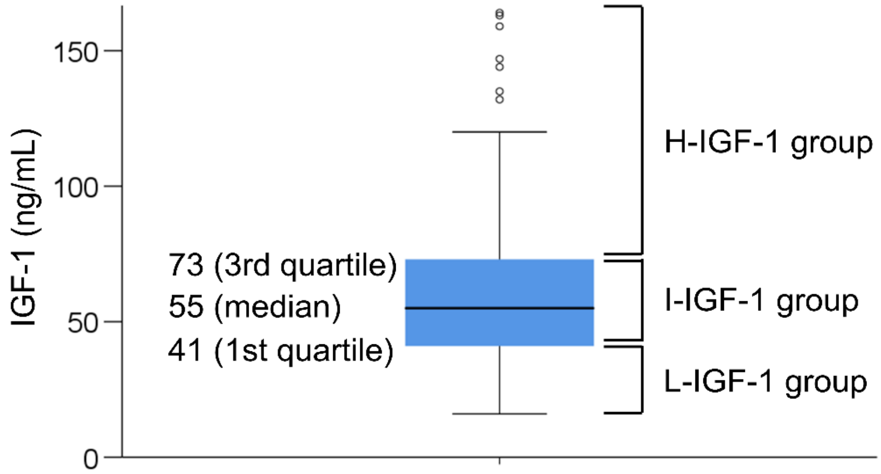

Figure S2. Classification based on baseline serum insulin-like growth factor 1 (IGF-1) levels. The median (interquartile range) of the IGF-1 levels was 55 (41–73) ng/mL. The 192 patients were classified into three groups: (1) low (L)-IGF-1 group with IGF-1 levels ≤41 ng/mL (first quartile); (2) intermediate (I)-BCAA group with IGF-1 levels between 41 and 73 ng/mL (third quartile); and (3) high (H)-IGF-1 group with IGF-1 levels ≥73 ng/mL.

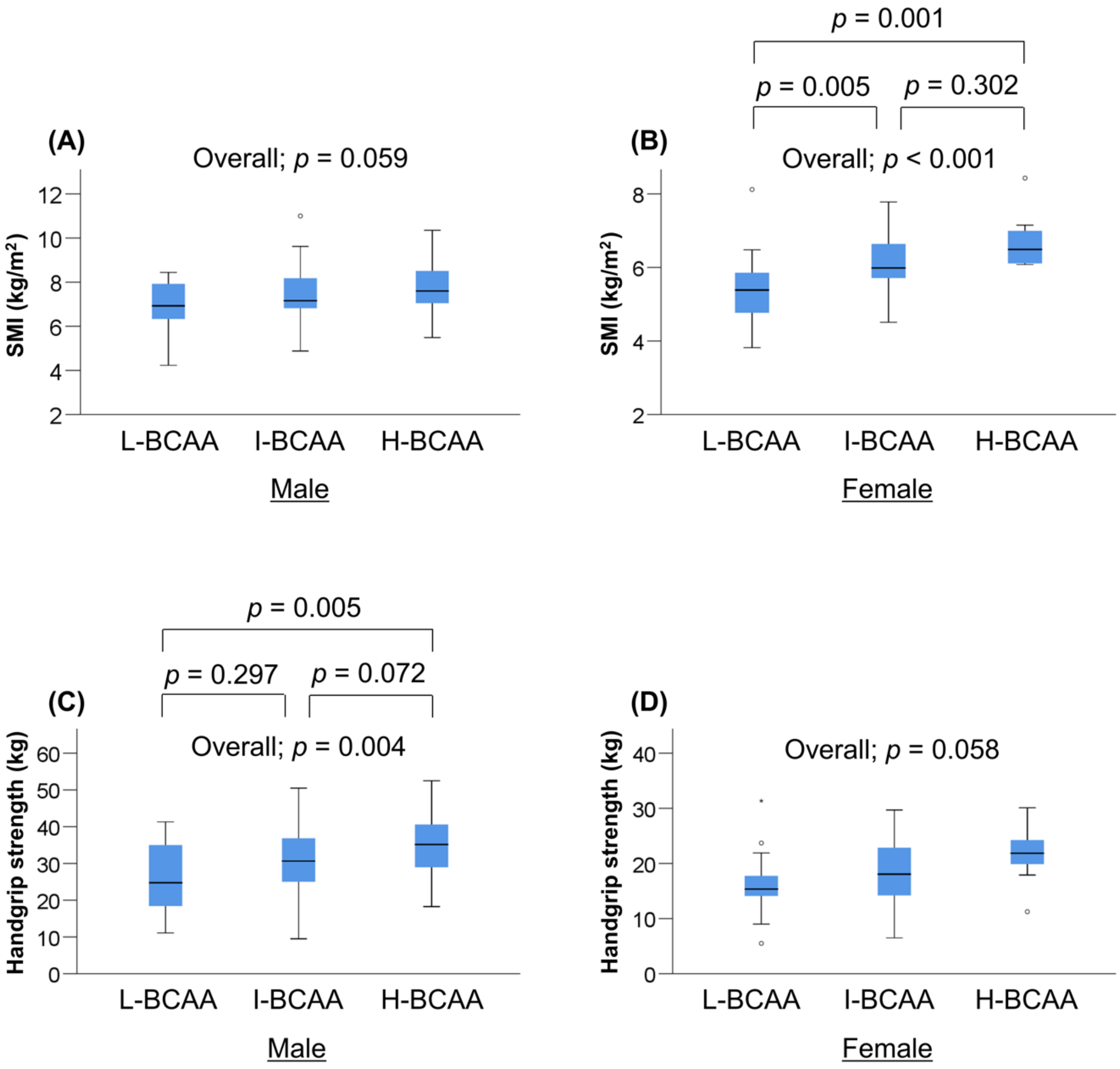

Figure S3. Comparison of clinical characteristics among the low (L)-branched-chain amino acid (BCAA), intermediate (I)-BCAA, and H-BCAA groups according to gender. (**A**) Skeletal muscle mass index (SMI) decreased stepwise with a reduction in the BCAA level in male patients, although the difference was not statistically significant. (**B**) SMI values were significantly lower in the L-BCAA group than in the I-BCAA and H-BCAA groups in female patients. (**C**) Handgrip strength values were significantly lower in the L-BCAA group than in the H-BCAA group in male patients. (**D**) Handgrip strength decreased stepwise with a reduction in the BCAA level in female patients, although the difference was not statistically significant.

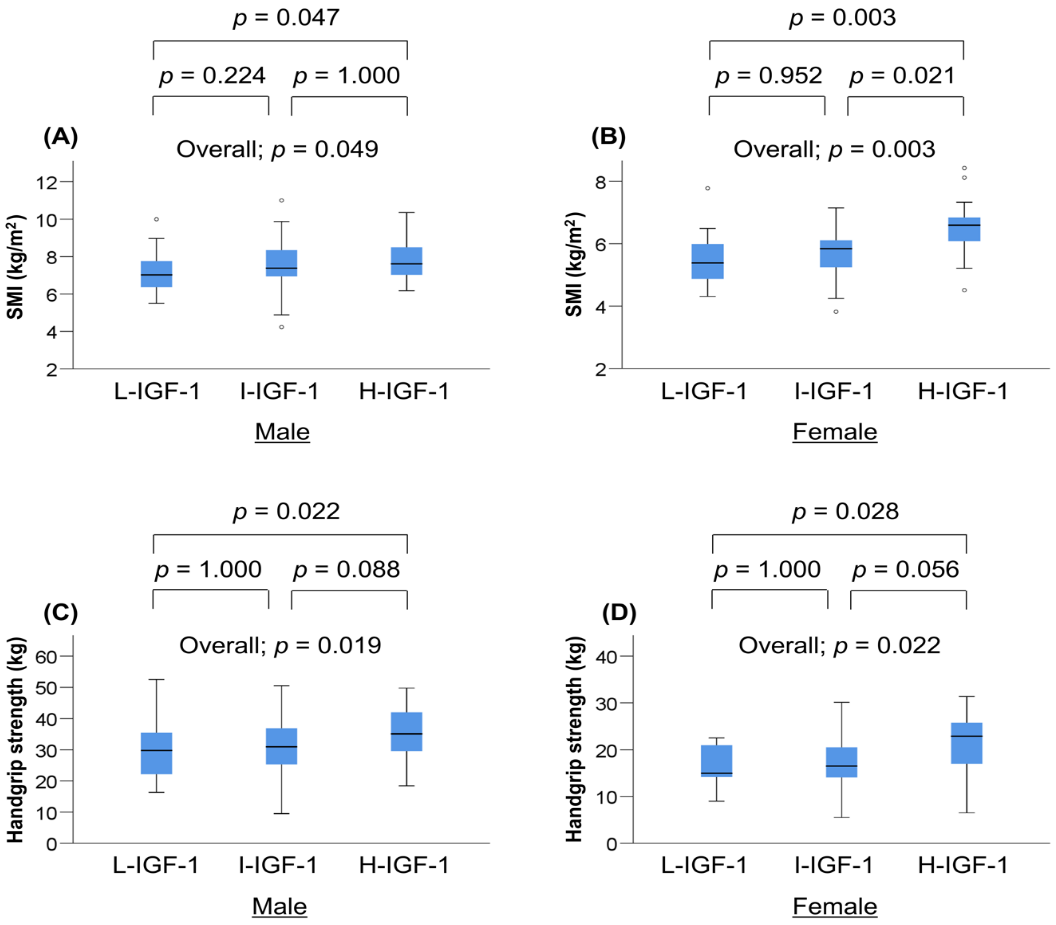

Figure S4. Comparison of clinical characteristics among the low (L)-insulin-like growth factor 1 (IGF-1), I-IGF-1, and H-IGF-1 groups according to gender. (**A**), (**C**) Skeletal muscle mass index (SMI) and handgrip strength values were significantly lower in the L-IGF-1 group than in the H-IGF-1 group in male patients. (**B**) SMI values were significantly lower in the L-IGF-1 and I-IGF-1 groups than in the H-IGF-1 group in female patients. (**D**) Handgrip strength values were significantly lower in the L-IGF-1 group than in the H-IGF-1 group in female patients.

Table S1. Univariate analysis for factors associated with sarcopenia.

Variable	OR (95% CI)	*p* Value
Age (years)	1.079 (1.041–1.117)	<0.001
Male	0.574 (0.299–1.099)	0.094
BMI (kg/m^2)	0.707 (0.622–0.803)	<0.001
Etiology	0.750 (0.534–1.055)	0.099
Child–Pugh B + C	1.249 (0.647–2.410)	0.507
Total bilirubin (mg/dL)	1.219 (0.813–1.828)	0.337
Albumin (g/dL)	0.470 (0.267–0.826)	0.009
Prothrombin time (%)	1.005 (0.985–1.025)	0.625
IGF-1 (ng/mL)	0.960 (0.942–0.978)	<0.001
BCAA (μmol/L)	0.989 (0.985–0.993)	<0.001
Zinc (μg/dL)	0.984 (0.964–1.005)	0.132
SMI (kg/m^2)	0.172 (0.101–0.292)	<0.001
Handgrip strength (kg)	0.829 (0.782–0.878)	<0.001

BCAA, branched-chain amino acid; BMI, body mass index; CI, confidence interval; IGF-1, insulin-like growth factor 1; OR, odds ratio; SMI, skeletal muscle mass index.

Table S2. Correlation between serum BCAA concentrations and baseline characteristics.

Variable	Correlation Coefficient	*p* Value
Age (years)	0.075	0.303
BMI (kg/m^2)	0.305	<0.001
Child–Pugh score	−0.341	<0.001
Total bilirubin (mg/dL)	0.013	0.862
Albumin (g/dL)	0.317	<0.001
Prothrombin time (%)	0.238	0.001
IGF-1 (ng/mL)	0.293	<0.001
Zinc (μg/dL)	0.307	<0.001
SMI (kg/m^2)	0.409	<0.001
Handgrip strength (kg)	0.419	<0.001
Gait speed (m/s)	0.245	0.001

BCAA, branched-chain amino acid; BMI, body mass index; IGF-1, insulin-like growth factor 1; SMI, skeletal muscle mass index.

Table S3. Correlation between serum IGF-1 concentrations and baseline characteristics.

Variable	Correlation Coefficient	*p* Value
Age (years)	−0.129	0.075
BMI (kg/m^2)	0.192	0.008
Child–Pugh score	−0.253	<0.001
Total bilirubin (mg/dL)	0.174	0.016
Albumin (g/dL)	0.270	<0.001
Prothrombin time (%)	0.305	<0.001
BCAA (μmol/L)	0.293	<0.001
Zinc (μg/dL)	0.176	0.016
SMI (kg/m^2)	0.261	<0.001
Handgrip strength (kg)	0.256	<0.001
Gait speed (m/s)	0.355	<0.001

BCAA, branched-chain amino acid; BMI, body mass index; IGF-1, insulin-like growth factor 1; SMI, skeletal muscle mass index.
